# Electrokinetic and Electroconvective Effects in Ternary Electrolyte Near Ion-Selective Microsphere

**DOI:** 10.3390/membranes13050503

**Published:** 2023-05-10

**Authors:** Georgy S. Ganchenko, Maxim S. Alekseev, Ilya A. Moroz, Semyon A. Mareev, Vladimir S. Shelistov, Evgeny A. Demekhin

**Affiliations:** 1 Laboratory of Micro- and Nanoscale Electro- and Hydrodynamics, Financial University under the Government of the Russian Federation, 53 Leningradsky Prospect str., Moscow 125167, Russia; 2 Membrane Institute, Kuban State University, 149 Stavropolskaya str., Krasnodar 350040, Russia; 3 Laboratory of General Aeromechanics, Institute of Mechanics, Moscow State University, 1 Michurinsky Prospect, Moscow 119192, Russia

**Keywords:** electrophoresis, ion-selective surface, ternary electrolyte, numerical modeling

## Abstract

The paper presents theoretical and experimental investigations of the behavior of an electrolyte solution with three types of ions near an ion-selective microparticle with electrokinetically and pressure-driven flow. A special experimental cell has been developed for the investigations. An anion-selective spherical particle composed of ion-exchange resin is fixed in the center of the cell. An enriched region with a high salt concentration appears at the anode side of the particle when an electric field is turned on, according to the nonequilibrium electrosmosis behavior. A similar region exists near a flat anion-selective membrane. However, the enriched region near the particle produces a concentration jet that spreads downstream akin to a wake behind an axisymmetrical body. The fluorescent cations of Rhodamine-6G dye are chosen as the third species in the experiments. The ions of Rhodamine-6G have a 10-fold lower diffusion coefficient than the ions of potassium while bearing the same valency. This paper shows that the concentration jet behavior is described accurately enough with the mathematical model of a far axisymmetric wake behind a body in a fluid flow. The third species also forms an enriched jet, but its distribution turns out to be more complex. The concentration of the third species increases in the jet with an increase in pressure gradient. The pressure-driven flow stabilizes the jet, yet electroconvection has been observed near the microparticle for sufficiently strong electric fields. The electrokinetic instability and the electroconvection partially destroy the concentration jet of salt and the third species. The conducted experiments show good qualitative agreement with the numerical simulations. The presented results could be used in future for implementing microdevices based on membrane technology for solving problems of detection and preconcentration, and thus simplifying chemical and medical analyses utilizing the superconcentration phenomenon. Such devices are called membrane sensors, and are actively being studied.

## 1. Introduction

The application of microfluidic technologies presents a very promising direction for prospective medical diagnostic systems [1,2,3]. However, the introduction of such technologies is associated with the problem of low analyte concentrations in human biological liquids. It is often necessary to pre-concentrate the analyte in the proximity of a microsensor to obtain sufficient sensing accuracy. Ion-selective surfaces play a key role in attempts to solve this problem [4,5], since the effect of concentration polarization can occur near such surfaces under the external electric field [6,7,8]. Concentration polarization allows controlling both the electrolyte as a whole and its individual components: ions and other suspended particles. At the same time, it is known [9,10,11] that the phenomenon of concentration polarization at sufficiently strong external electric fields can lead to electrokinetic instability and generate complex nonstationary electroconvective regimes, up to stochastic ones [12,13,14,15,16]. This phenomenon is actively used in microfluidic devices based on ion-exchange membranes and/or granules [17] for sequencing macromolecules (including DNA), for separation of dispersed particles by size and electrical conductivity, for creating new biomaterials, in micro-pumps and micromixers, etc. [18]. The electroconvection makes it difficult to conduct experimental studies without in-depth theoretical analysis. In this regard, mathematical modeling plays a significant role in the study of the processes described above, as it provides a detailed analysis with relatively small effort.

In the current literature, two qualitatively different schemes of pre-concentration of the electrolyte can be distinguished. The first concept is based on using a complex planar system of channels, ion-exchange membranes and electrodes [5,19]. The second concept of the device design is based on using a spherical geometry [20,21]. The first approach is characterized by a high degree of analyte concentration, and the second one is distinguished by simplicity and versatility, because in such geometry one device can simultaneously perform the roles of a micro-pump [22], a micromixer [20,23], a microreactor, and a microseparator [21].

In this paper, mathematical modeling and an experimental study of the analyte concentration in a microdevice based on an ion-selective microsphere will be presented. Within the framework of our study, spherical membranes may be used in the future for creating microdevices. One potential application of the geometry we are studying is connected to the problems of preconcentration and detection in membrane technology, which are atypical in this field but have nevertheless been actively researched recently [5]. Such devices are known as membrane sensors, which can significantly simplify chemical and medical analyses by utilizing the effect of superconcentration [1]. In research carried out by [1,5], the authors considered the use of flat membranes; however, our study focuses on a simpler formulation to examine the nature of the superconcentration effect. Superconcentration effect in an identical system has been experimentally studied before [24] but for an AC field, however, the difference in mobility and ion charge still remains the main source of the superconcentration effect. Membrane systems are known to split water at overlimiting conditions [25]. However, such conditions usually occur at high electric current regimes, which are beyond the scope of our current research. This is because overlimiting regimes are known to initiate instabilities which can lead to liquid mixing and consequently, lower concentration levels. In the future, we intend to evaluate the efficiency of using spherical membranes compared to their flat counterparts for membrane sensors.

## 2. Statement

### 2.1. Geometric Characteristics

**Figure 1:**
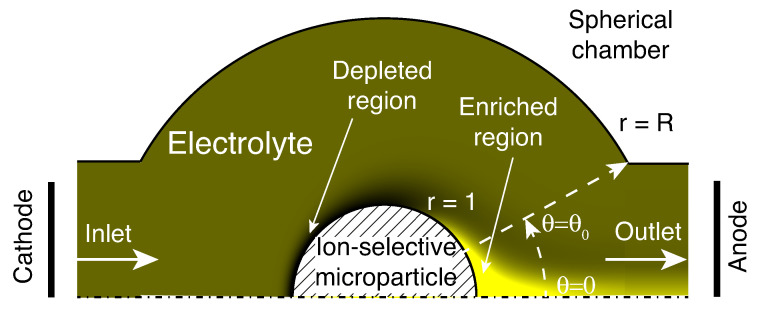
Schematic of the cell specifying the main areas near the anion-selective microgranule. The shades of yellow qualitatively specify a typical distribution of concentration.

This paper considers a cell consisting of a spherical hollow chamber made of a solid dielectric material with a round inlet and outlet (Figure 1). The cell is connected to external reservoirs filled with electrolyte, into which the electrodes are placed. The electrolyte contains cations and anions of a salt and charged species of an analyte. The inlet-side reservoir is connected to a pump that creates additional pressure. An ion-selective spherical particle is anchored to the center of the chamber. The method of particle anchoring in the experimental installation will be described in the corresponding section, while the mathematical model neglects its particular realization.

An axisymmetric formulation is considered in the mathematical model. The axis of symmetry connects the centers of the inlet and the outlet. The spherical chamber is assumed to be composed of an ideal dielectric with some uniform surface charge. The ion-selective particle is assumed to be partially selective, that is, allowing both counter- and co-ions to move through it. The hydrodynamics inside the particle are neglected.

The particle is assumed to be perfectly homogeneous, without any imperfections, thus it consists of the gel phase only. It should be mentioned that real resins may have macropores filled with uncharged solution. There are several modeling approaches to describe such systems [26]. In the current paper, the complex structure of a real ion-exchange particle is not taken into account for the sake of simplicity.

For the sake of certainty, the particle is assumed to be anion-selective. The influence of inlet and outlet channels is ignored: the influence of the electric field and additional pressure is described with constant of potential and pressure differences at the inlet and the outlet parts of the chamber.

### 2.2. Dimensional Formulation

The concentration process of ions and analyte is considered. Locally, the concentration density may exceed the initial one by at least one order of magnitude. Nevertheless, we have chosen a low enough range of the initial concentrations to meet a highly diluted electrolyte assumption [9]. This is also the case for the third species concentration, because the concentration of analytes in human liquids is several orders lower than the salt concentration [4]. In addition, the analyte concentration is not known to exceed maximum salt concentration density even for million-fold preconcentration [19].

The direct interaction of ions and chemical reactions are neglected. In this case, the behavior of the electrolyte is described by the system of Nernst–Planck equations with respect to the density of ion concentrations

(1) $$\frac{\partial {\tilde{C}}^{\pm }}{\partial \tilde{t}}+\tilde{U}\cdot \nabla {\tilde{C}}^{\pm }=\frac{z^{\pm }{\tilde{D}}^{\pm }\tilde{F}}{\tilde{R}\tilde{T}}\nabla \cdot \left({\tilde{C}}^{\pm }\nabla \tilde{\Phi }\right)+{\tilde{D}}^{\pm }\nabla^{2}{\tilde{C}}^{\pm }.$$

The presence of a third charged species is assumed in a salt electrolyte, which renders the electrolyte ternary.

(2) $$\frac{\partial {\tilde{C}}_{a}}{\partial \tilde{t}}+\tilde{U}\cdot \nabla {\tilde{C}}_{a}=\frac{z_{a}{\tilde{D}}_{a}\tilde{F}}{\tilde{R}\tilde{T}}\nabla \cdot \left({\tilde{C}}_{a}\nabla \tilde{\Phi }\right)+{\tilde{D}}_{a}\nabla^{2}{\tilde{C}}_{a}.$$

The system is supplemented by the Poisson equation on the electric potential and the Stokes equations on the velocity field.

(3) $$\tilde{\varepsilon }\nabla^{2}\tilde{\Phi }=-\tilde{F}\left(z^{+}{\tilde{C}}^{+}+z^{-}{\tilde{C}}^{-}+z_{a}{\tilde{C}}_{a}\right),$$

(4) $$\nabla \tilde{\Pi }-\tilde{\mu }\nabla^{2}\tilde{U}=-\tilde{F}\left(z^{+}{\tilde{C}}^{+}+z^{-}{\tilde{C}}^{-}+z_{a}{\tilde{C}}_{a}\right)\nabla \tilde{\Phi },$$

(5) $$\nabla \cdot \tilde{U}=0.$$

The unknowns are as follows: the molar concentration densities of the salt and the analyte ions, ${\tilde{C}}^{\pm }$, ${\tilde{C}}_{a}$, the electric potential, $\tilde{\Phi }$, the pressure, $\tilde{\Pi }$, and the velocity field, $\tilde{U}$. Here, $\tilde{F}$ is the Faraday number, $\tilde{R}$ is the universal gas constant, and $\tilde{T}$ is the absolute temperature. The variables denoted with tildes are dimensional. In contrast, the variables without tildes are dimensionless. The base electrolyte is assumed to be symmetric, i.e., it has the same diffusion coefficient, ${\tilde{D}}^{+}={\tilde{D}}^{-}=\tilde{D}$, and the same absolute value of the valences, $z^{+}=-z^{-}=1$, for cations and anions. $\tilde{\mu }$ is the dynamic viscosity of the electrolyte, $\tilde{\varepsilon }$ is its electrical permeability. The system of equations is solved in a spherical axisymmetric formulation. We will consider the case of $z_{a}=1$, so the only difference between salt ions and the analyte species is the diffusion coefficient of the latter, ${\tilde{D}}_{a}$.

The ions and the analyte impermeability condition is accepted on the surface of the outer dielectric sphere, $\tilde{r}={\tilde{r}}_{1}$, $\theta_{0}<\theta <\pi -\theta_{0}$,

(6) $$\frac{\partial {\tilde{C}}^{\pm }}{\partial \tilde{r}}+\frac{z^{\pm }\tilde{F}}{\tilde{R}\tilde{T}}{\tilde{C}}^{\pm }\frac{\partial \tilde{\Phi }}{\partial \tilde{r}}=0,\;\frac{\partial {\tilde{C}}_{a}}{\partial \tilde{r}}+\frac{z_{a}\tilde{F}}{\tilde{R}\tilde{T}}{\tilde{C}}_{a}\frac{\partial \tilde{\Phi }}{\partial \tilde{r}}=0,$$

where $\tilde{r}$ is the direction along the radius, centered in the middle of the ion-selective microgranule, and θ is the angle (Figure 1).

A charge is assumed to be present on the surface of the spherical chamber, which makes it possible to establish a boundary condition for the electric potential $\tilde{\Phi }$,

(7) $$\tilde{\varepsilon }\frac{\partial \tilde{\Phi }}{\partial \tilde{r}}=-\tilde{\sigma }.$$

The no-slip condition applies to the spherical chamber,

(8) $$\tilde{U}=0.$$

The reservoir-type boundary conditions for the molar ionic concentration are given together with the boundary conditions for pressure and electric potential on the outlet is $\tilde{r}={\tilde{r}}_{1}$, $0<\theta <\theta_{0}$ (see Figure 1),

(9) $$\frac{\partial {\tilde{C}}^{\pm }}{\partial \tilde{r}}=0,\;\frac{\partial {\tilde{C}}_{a}}{\partial \tilde{r}}=0,\;\tilde{\Pi }=0,\;\tilde{\Phi }=\Delta \tilde{V}/2.$$

The salt concentration, the pressure, and the electric potential values are fixed at the inlet, $\tilde{r}={\tilde{r}}_{1}$, $\pi -\theta_{0}<\theta <\pi$,

(10) $${\tilde{C}}^{+}={\tilde{C}}_{\infty },\;{\tilde{C}}_{a}={\tilde{C}}_{a}^{0},\;z^{+}{\tilde{C}}^{+}+z^{-}{\tilde{C}}^{-}+z_{a}{\tilde{C}}_{a}=0,\;\tilde{\Pi }=\Delta \tilde{\Pi },\;\tilde{\Phi }=-\Delta \tilde{V}/2.$$

The electrolyte flow is both electrokinetically and pressure-driven. The corresponding forces are controlled with $\Delta \tilde{V}$ and $\Delta \tilde{\Pi }$, respectively.

The Equations (1) and (2) are also solved inside the ion-selective particle $\tilde{r}<{\tilde{r}}_{0}$, where the Poisson equation takes the form

(11) $$\tilde{\varepsilon }\nabla^{2}\tilde{\Phi }=-\tilde{F}\left(z^{+}{\tilde{C}}^{+}+z^{-}{\tilde{C}}^{-}+z_{a}{\tilde{C}}_{a}\right)+\tilde{F}\tilde{N}.$$

This equation includes a uniform volume charge density of $\tilde{N}$ that controls the selectivity of the particle. A cation-selective particle has $\tilde{N}>0$, an anion-selective one has $\tilde{N}<0$. Higher absolute values of $\tilde{N}$ correspond to higher ion selectivity. Introducing $\tilde{N}$ in (11) is a simple and effective way for numerical simulation of imperfectly selective membranes [27]. Physically, the volume charge is associated with the ion-exchange capacity.

The hydrodynamics inside the particle were neglected, $\tilde{U}\equiv 0$. A similar method of modeling imperfect selective surfaces has been used in solving problems in planar geometry [28] and proven to be a good approximation for generalizing the model relative to the perfectly selective approach [9,11,29,30,31].

### 2.3. Dimensionless Formulation

The following characteristic values have been used to make Equations (1)–(11) dimensionless.

The Equations (1)–(5) in the dimensionless form and in the axisymmetrical spherical coordinates are as follows. Equation (1) for the ion transport turns into,

(12) $$\begin{array}{c} \frac{\partial C^{+}}{\partial t}+U\frac{1}{r}\frac{\partial C^{+}}{\partial \theta }+V\frac{\partial C^{+}}{\partial r}=\left[\frac{1}{r^{2}\sin\theta }\frac{\partial }{\partial \theta }\left(\sin\theta C^{+}\frac{\partial \Phi }{\partial \theta }\right)+\frac{1}{r^{2}}\frac{\partial }{\partial r}\left(r^{2}C^{+}\frac{\partial \Phi }{\partial r}\right)\right]+\\ +\left[\frac{1}{r^{2}\sin\theta }\frac{\partial }{\partial \theta }\left(\sin\theta \frac{\partial C^{+}}{\partial \theta }\right)+\frac{1}{r^{2}}\frac{\partial }{\partial r}\left(r^{2}\frac{\partial C^{+}}{\partial r}\right)\right], \end{array}$$

(13) $$\begin{array}{c} \frac{\partial C^{-}}{\partial t}+U\frac{1}{r}\frac{\partial C^{-}}{\partial \theta }+V\frac{\partial C^{-}}{\partial r}=-\left[\frac{1}{r^{2}\sin\theta }\frac{\partial }{\partial \theta }\left(\sin\theta C^{-}\frac{\partial \Phi }{\partial \theta }\right)+\frac{1}{r^{2}}\frac{\partial }{\partial r}\left(r^{2}C^{-}\frac{\partial \Phi }{\partial r}\right)\right]+\\ +\left[\frac{1}{r^{2}\sin\theta }\frac{\partial }{\partial \theta }\left(\sin\theta \frac{\partial C^{-}}{\partial \theta }\right)+\frac{1}{r^{2}}\frac{\partial }{\partial r}\left(r^{2}\frac{\partial C^{-}}{\partial r}\right)\right], \end{array}$$

(14) $$\begin{array}{c} \frac{\partial C_{a}}{\partial t}+U\frac{1}{r}\frac{\partial C_{a}}{\partial \theta }+V\frac{\partial C_{a}}{\partial r}=D_{a}\left[\frac{1}{r^{2}\sin\theta }\frac{\partial }{\partial \theta }\left(\sin\theta C_{a}\frac{\partial \Phi }{\partial \theta }\right)+\frac{1}{r^{2}}\frac{\partial }{\partial r}\left(r^{2}C_{a}\frac{\partial \Phi }{\partial r}\right)\right]+\\ +D_{a}\left[\frac{1}{r^{2}\sin\theta }\frac{\partial }{\partial \theta }\left(\sin\theta \frac{\partial C_{a}}{\partial \theta }\right)+\frac{1}{r^{2}}\frac{\partial }{\partial r}\left(r^{2}\frac{\partial C_{a}}{\partial r}\right)\right], \end{array}$$

the Poisson Equation (3) is now presented by the following equation,

(15) $$\frac{\nu^{2}}{r^{2}}\left[\frac{\partial }{\partial r}\left(r^{2}\frac{\partial \Phi }{\partial r}\right)+\frac{1}{\sin\theta }\frac{\partial }{\partial \theta }\left(\sin\theta \frac{\partial \Phi }{\partial \theta }\right)\right]=C^{-}-C^{+}-C_{a}\equiv -\rho ,$$

outside the particle (1<r<R) and

(16) $$\frac{\nu^{2}}{r^{2}}\left[\frac{\partial }{\partial r}\left(r^{2}\frac{\partial \Phi }{\partial r}\right)+\frac{1}{\sin\theta }\frac{\partial }{\partial \theta }\left(\sin\theta \frac{\partial \Phi }{\partial \theta }\right)\right]=C^{-}-C^{+}-C_{a}+N,$$

inside the particle (r<1). The Stokes Equations (4) and (5) for creeping flow turn into the following ones,

(17) $$-\frac{1}{r}\frac{\partial \Pi }{\partial \theta }+\frac{\partial^{2}U}{\partial r^{2}}+\frac{2}{r}\frac{\partial U}{\partial r}+\frac{1}{r^{2}}\frac{\partial^{2}U}{\partial \theta^{2}}+\frac{\cot\theta }{r^{2}}\frac{\partial U}{\partial \theta }-\frac{U}{r^{2}\sin^{2}\theta }+\frac{2}{r^{2}}\frac{\partial V}{\partial \theta }=\rho \frac{1}{r}\frac{\kappa }{\nu^{2}}\frac{\partial \Phi }{\partial \theta },$$

(18) $$-\frac{\partial \Pi }{\partial r}+\frac{\partial^{2}V}{\partial r^{2}}+\frac{2}{r}\frac{\partial V}{\partial r}+\frac{1}{r^{2}}\frac{\partial^{2}V}{\partial \theta^{2}}-\frac{2V}{r^{2}}+\frac{\cot\theta }{r^{2}}\frac{\partial V}{\partial \theta }-\frac{2U}{r^{2}}\cot\theta -\frac{2}{r^{2}}\frac{\partial U}{\partial \theta }=\rho \frac{1}{r}\frac{\kappa }{\nu^{2}}\frac{\partial \Phi }{\partial r},$$

(19) $$\frac{\partial }{\partial \theta }\left(\sin\theta \;r\;U\right)+\frac{\partial }{\partial r}\left(\sin\theta \;r^{2}\;V\right)=0,$$

in the electrolyte (1<r<R). Here, (U,V) are the velocity components. The dimensionless parameter ν is the Debye number, which is the ratio of the Debye length ${\tilde{\lambda }}_{D}$ and the microgranule radius ${\tilde{r}}_{0}$ (ν≪1 is a small parameter of the problem, a thin electric double layer (EDL) is considered),

$$\nu =\frac{{\tilde{\lambda }}_{D}}{{\tilde{r}}_{0}},\;{\tilde{\lambda }}_{D}={\left(\frac{\tilde{\varepsilon }{\tilde{\Phi }}_{0}}{\tilde{F}{\tilde{C}}_{\infty }}\right)}^{\frac{1}{2}},$$

and κ is the coupling coefficient between the hydrodynamics and the electrostatics,

$$\kappa =\frac{\tilde{\varepsilon }{\tilde{\Phi }}_{0}^{2}}{\tilde{\mu }\tilde{D}}.$$

This quantity characterizes the physical properties of the electrolyte solution and is fixed for a given liquid and electrolyte. The value of ν depends on two main factors. The first one is ${\tilde{r}}_{0}$: ν decreases with increasing the characteristic length ${\tilde{r}}_{0}$. The second one is ${\tilde{C}}_{\infty }$: ν decreases with increasing the characteristic concentration ${\tilde{C}}_{\infty }$. This means that ν is different for highly diluted and moderately diluted electrolytes. Basically, a highly diluted electrolyte is considered in this paper.

The conditions on the outer dielectric sphere, $r=R={\tilde{r}}_{1}/{\tilde{r}}_{0}$, $\theta_{0}<\theta <\pi -\theta_{0}$, Equations (6)–(8), turn into

(20) $$\frac{\partial C^{\pm }}{\partial \tilde{r}}\pm C^{\pm }\frac{\partial \Phi }{\partial r}=0,\frac{\partial C_{a}}{\partial \tilde{r}}+C_{a}\frac{\partial \Phi }{\partial r}=0,$$

(21) $$\nu \frac{\partial \Phi }{\partial r}=-\sigma ,$$

(22) U=0,

where $\sigma =\tilde{\sigma }{\tilde{\lambda }}_{D}/\tilde{\varepsilon }{\tilde{\Phi }}_{0}$ is the dimensionless surface charge.

At the outlet, r=R, $0<\theta <\theta_{0}$, the conditions (9) are now rewritten in a dimensionless form,

(23) $$\frac{\partial C^{\pm }}{\partial r}=0,\;\frac{\partial C_{a}}{\partial r}=0,\;\Pi =0,\;\Phi =\Delta V/2,$$

where ΔV/2 is the dimensionless potential at the outlet.

At the inlet, r=R, $\pi -\theta_{0}<\theta <\pi$, the salt concentration distribution along the hole, the electroneutrality condition, the pressure and the electric potential are given,

(24) $$C^{+}=1,\;C_{a}=C_{a}^{0},\;C^{-}=1+C_{a},\;\Pi =\Delta \Pi ,\;\Phi =-\Delta V/2.$$

These conditions are the dimensionless version of Equations (10). The potential drop $\Delta V=\Delta \tilde{V}/{\tilde{\Phi }}_{0}$ and the pressure difference $\Delta \Pi =\Delta \tilde{\Pi }{\tilde{r}}_{0}^{2}/\tilde{\mu }\tilde{D}$ may have different signs, they may be either co- or counter-directed. The direction depends on a vertical orientation of the device. They give rise to the electroosmotic and the pressure-driven flow, respectively.

Thus, the system has two geometric dimensional parameters, $R={\tilde{r}}_{1}/{\tilde{r}}_{0}$ and $\theta_{0}$. The first parameter characterizes the channel width, and the second parameter characterizes the size of the inlet and outlet. The properties of an ion-selective particle are described by the parameter $N=\tilde{N}/{\tilde{C}}_{\infty }$, which is associated with the capacity of the ion-exchange material from which the particle is made. The properties of the third species of ions are described by a single parameter $D_{a}={\tilde{D}}_{a}/\tilde{D}$. For example, ${\tilde{D}}_{a}$ is about $2\times 10^{-10}$ m^2^/s for Rhodamine-6G cations. The initial concentration of the the third species is fixed at $C_{a}^{0}=0.1$.

Typical dimensional quantities are chosen equal or close to those that have been reproduced in the experiment. The characteristic radius of a micromolecule ${\tilde{r}}_{0}$ is 350 µm, the typical diffusion coefficients $\tilde{D}$ for potassium and chlorine ions are about, $\tilde{D}∼2\times 10^{-9}$ m^2^/s, the characteristic density of ion concentration is ${\tilde{C}}_{\infty }=0.05$ mol/m^3^, the thermal potential at $\tilde{T}=300$ K is approximately ${\tilde{\Phi }}_{0}=25$ mV. Highly diluted aqueous solutions of electrolytes are considered, therefore, the parameters of the dynamic viscosity and the permittivity for pure water have been taken, $\tilde{\mu }=9\times 10^{-4}$ Pa·s, $\tilde{\varepsilon }=7\times 10^{-10}$ C/V·m.

The dimensional voltage varied from 0 to $\Delta \tilde{V}=2.5$ V. The value of the pressure gradient ΔΠ was adjusted to reach the flowrate from 0.04 mL/min to 0.12 mL/min. Taking into account the geometric characteristics, the calculations have showed that the range of ΔΠ from 1500 to 4500 corresponds to such flowrates.

The surface charge density of the dielectric surfaces $\tilde{\sigma }$ varies widely for different types of materials. For example, the surface charge density of quartz glass is between $\tilde{\sigma }=10^{-4}$ C/m^2^ to $\tilde{\sigma }=10^{-3}$ C/m^2^. A plastic for 3D printing and PMMA have been used in the experiment, their surface charge values are not well investigated. We have fixed the dimensionless surface charge at σ=1 for our calculations; the simulations for other values of σ show that its influence on the processes is weak. The radius of the outer sphere and the angle of the entrance section were fixed, R=3 and $\theta_{0}=30^{\circ }$. The parameters κ and ν were also fixed, κ=0.2 (which roughly corresponds to a potassium chloride solution) and $\nu =5\times 10^{-4}$. The value of *N* was fixed at N=−10, which corresponds to a selectivity of more than 90% [28].

### 2.4. Numerical Method

The system of Equations (12)–(24) has a small parameter, the Debye number, at the highest-order derivatives. As a result, there is a thin charged region with a rapid change of the unknown functions near the surface. This causes significant difficulties in seeking a numerical solution for the problem. These difficulties are compounded by the complexity of the chaotic regime when the flow contains a wide range of different scales. There are two main approaches to overcome these difficulties. The first one is a semi-analytical approach, where the solution in the Debye layer is sought analytically as the inner expansion, leaving the numerics for the diffusion region that is treated as the outer expansion. Of course, a proper matching of the inner and outer expansions is needed. This method has been systematically applied for charged dielectric particles by Yariv’s group (see, for example [32]); it has also been used for ion-selective granules in [30]. The second approach solves the entire system of Nernst–Planck–Poisson–Stokes equations numerically, without any simplification, and relies on sophisticated tuning of the numerical scheme instead.

The problem has been solved numerically using the finite difference method on a nonuniform grid for discretization in spatial variables *r* and θ. Time integration has been carried out using a semi-implicit method. Details of the numerical simulation method can be found in the papers [30,31]. The only difference is that for the present problem we do not need to use the force balance equation to obtain the microparticle velocity, because the particle is fixed. The ideas of the algorithm have been previously described in [33] for a planar statement, except that that paper uses FFT rather than Legendre/Gegenbauer polynomials. We rely on a step control subroutine [34] that ensures that the local error remains constrained by an externally specified parameter. Most of our simulations use local error 10${}^{-3}$, $\Delta t=10^{-8},\Delta \theta =0.0123$ and Δr varying from $3\times 10^{-4}$ to $3\times 10^{-2}$. We have verified the most important calculations by doubling the number of spatial grid points and by specifying a lower error bound.

### 2.5. Experimental Materials and Methods

**Figure 2:**
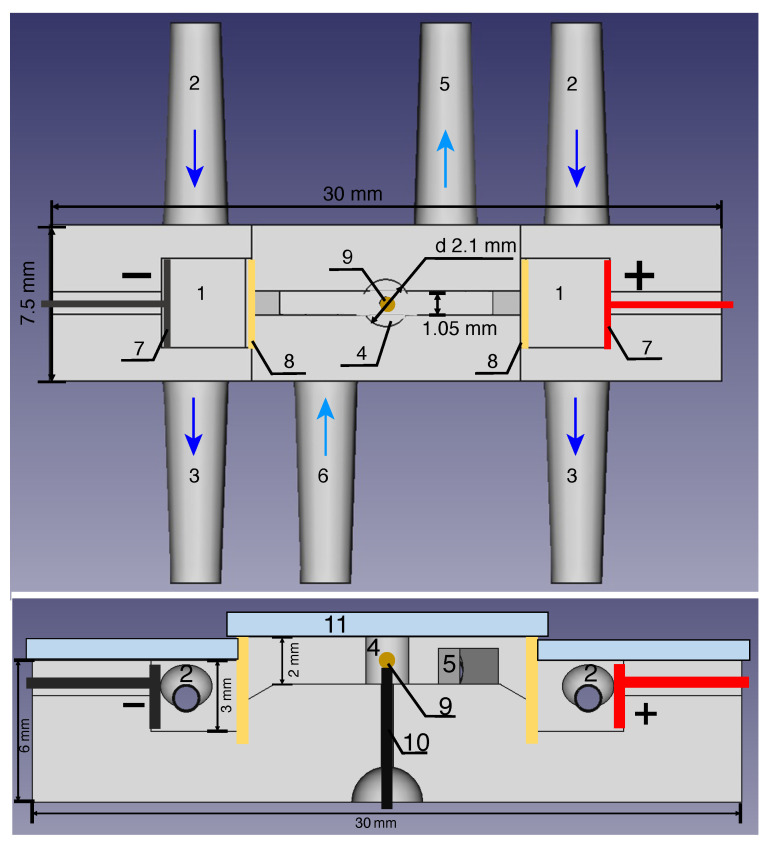
Schematic of the experimental cell: 1—the electrode chambers, 2 and 3—inlets and outlets of the electrode chambers, respectively, 4—the cylindrical chamber, 5—the output connector, 6—the input connector, 7—the electrodes, 8—the membranes, 9—the anion-selective particle, 10—the wire for fixing the particle, 11—the transparent PMMA cover. The arrows show the flow directions.

The design of the experimental cell differs in certain aspects from the numerical model. Visualization in a spherical chamber is a painstaking problem due to the need to use spherical lenses and take into account optical aberrations. A cylindrical chamber has been used instead of a spherical one to simplify the task (Figure 2). The ion-selective microgranule has been mounted at a thin wire inserted through a special hole at the center of the chamber. A thin layer of cyanocrylate adhesive was used for attaching ion-selective particle to the kernel to anchor it. The influence of the kernel on the concentrations is minimal due to insignificant size of kernel and adhesive layer compared to particle.

We have also used OPMN-P membranes (ZAO STC “Vladipor”, Vladimir, Russia) to prevent the bubbles from entering the cylindrical chamber. Detachable reservoirs have been used in addition to electrode chambers. It has been made possible to pump the electrolyte individually through each section and rinse the electrode chambers from by-products that inevitably form on electrodes during experiment. Large amounts of these by-products have the potential to significantly reduce the system’s operating surface, leading to decreased current. Moreover, the by-products can contaminate the main cylindrical chamber and thus affect the comparison with theoretical results. It is especially important to eliminate the by-products for limiting and overlimiting regimes, when their generation is highest.

The lower part of the experimental cell has been constructed through photopolymer printing on a 3D printer AnyCubic Photon (Shenzhen Anycubic Technology Co., Ltd., Shenzhen, China). The upper part was a 0.9 mm PMMA cover with high transparency to enable visualization.

**Figure 3:**
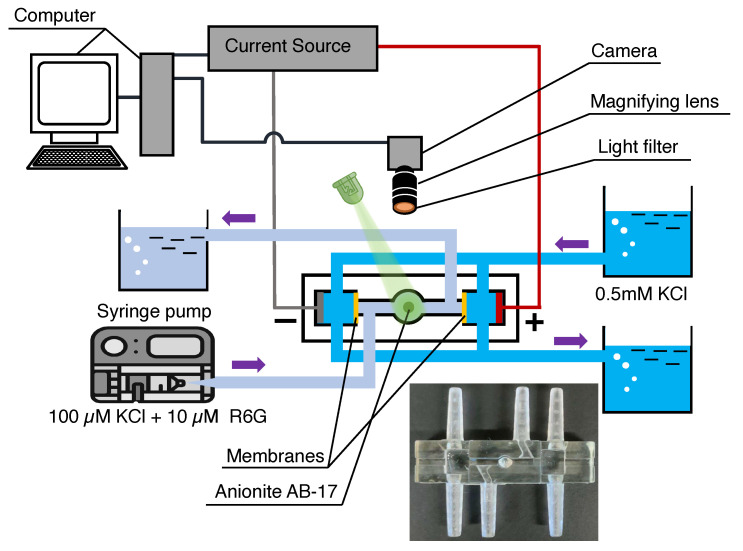
Schematic of the experimental set-up.

The fluorescent agent Rhodamine-6G (reagent grade, LenReaktiv, Saint-Peterburg, Russia) has been used as an analyte, which was diluted in a buffer solution of potassium chloride (analytical grade, LenReaktiv, Saint-Peterburg, Russia). In our model, potassium chloride corresponds to the salt dissociating into cations (K^+^) and anions (Cl^−^), and cations of Rhodamine-6G correspond to the macromolecule/third species of ions in the solution (Figure 3). The concentration of Rhodamine-6G (10 µM) in the cylindrical chamber was significantly lower than that of potassium chloride (100 µM). The concentration of the salt in the electrode chambers has been kept higher than in the cylindrical chamber in order to reduce the resistance of the system. The accuracy of the preparation of solutions was achieved by weighing salts on laboratory scales with the accuracy of 10${}^{-4}$ g. The driving force for moving the liquid through the chamber has been created by the syringe pump Instilar Dixion 1428. The electrolyte flowrate varied from 0.04 mL/min to 0.16 mL/min in the cylindrical chamber; it was raised to 5 mL/min in the electrode chambers. The anion-selective particle was Anionite AV-17 with 762 ± 5 µm diameter. A potential drop has been created by the Keithley 2400 SourceMeter current source. The Rhodamine molecules were excited by an LED light source with an emitted wavelength of 490 nm and re-emitted light in the wavelength range of 530–570 nm. The visualization of dye behavior was achieved using an optical microscope consisting of the camera TOUPCAM U3CMOS1800KPA with 20 frames-per-second framerate and a magnifying lens. Color post-processing was carried out using the RisingView software.

## 3. Results and Discussion

The behavior of the electrolyte near the ion-selective microparticle exhibits a number of bifurcations with an increase in the external electric field strength. Electrosmosis of the first kind is realized at low voltages, when the charge is located in a thin electric double layer [35]. This regime is typical for dielectric surfaces and particles [36]. With an increase in the external electric field strength, electroosmosis of the second kind [37] appears: it is characterized by complex nonlinear modes [30], instabilities and bifurcations [31].

### 3.1. Steady-State Regimes

First, the stationary regimes, ∂/∂t=0, are considered. A complex system of layers appears near the particle in the stationary regime. A special attention will be paid to the depleted region (which occurs near the cathode side for anion-selective membranes) and the enriched region (which occurs at the anode side). The latter is characterized by an increased salt concentration—several times higher than in the bulk solution.

The nature of the depleted region, which also includes the space charge region, has been studied in detail in problems with a flat-ion-selective surface [9,10,11]. This region appears due to concentration polarization. It is important to note that when the space charge region expands with an increase in the electric field strength, the influence of the surface curvature begins to significantly affect the flow inside this zone, so some asymptotic approximations made for a flat membrane [38] become invalid.

The enriched region on the other side of the particle is focused in the form of a fine structure that spreads downstream. Because of this behavior, we will refer to it as a concentration jet. For the case of an electroneutral jet, its propagation downstream can be described qualitatively by the problem of an axisymmetric wake behind a body of revolution in an axial flow [39],

(25) $$U_{\infty }\frac{\partial K}{\partial x}=\frac{1}{y}\frac{\partial }{\partial }\left(y\frac{\partial K}{\partial y}\right),$$

where $U_{\infty }$ is a mean velocity along the axis of symmetry, *x* is a Cartesian coordinate along the axis of symmetry, *y* is a Cartesian coordinate normal to the axis of symmetry, $K=C^{+}+C^{-}+C_{a}$ is the density of salt concentration. The solution of Equation (25) is as follows,

(26) $$K=2\left(1+C_{a}^{0}\right)+\frac{C_{D}}{U_{\infty }}\;\frac{1}{x-x_{0}}\exp\left(-\frac{U_{\infty }y^{2}}{4x}\right),$$

where $C_{D}$ is a parameter related to the intensity of the concentration source, and $x_{0}$ is the coordinate of the location of the point source of salt. A more detailed analysis of this solution is presented in [40]. The thickness of the mixing layer expands downstream as $y_{\delta }=\sqrt{x/U_{\infty }}$, and this layer narrows with increasing $U_{\infty }$. This means that with an increase in the pumping of the electrolyte the concentration jet visually becomes narrower and retains its intensity longer downstream. It will be demonstrated further that such a dependence is also valid for the analyte. It can be assumed that $C^{+}\approx K/2$, taking into account the electroneutrality in the concentration jet region $C^{+}=C^{-}-C_{a}$ and the smallness of the analyte concentration.

**Figure 4:**
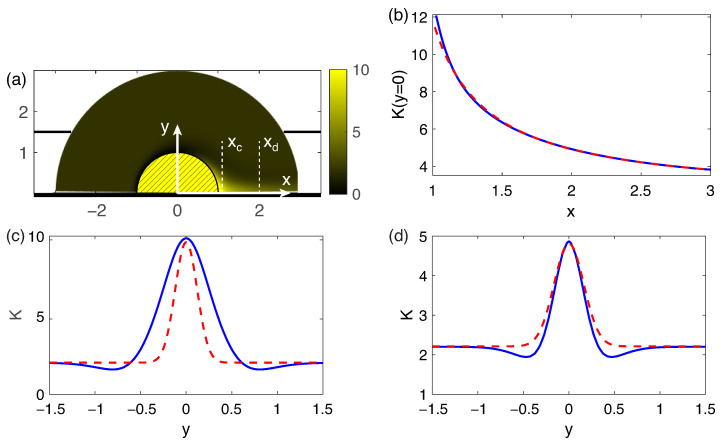
Densities of the salt concentration *K* at ΔV=100, ΔΠ=1500 (**a**), concentration density profiles for a numerical modeling (solid lines) and for the analytical Equation (26) (dashed lines) along the axis of symmetry (**b**) and in the sections $x_{c}=1.1$ (**c**) and $x_{d}=2$ (**d**), indicated in the picture (**a**).

The results of comparison of numerical simulation and analytical equation for ΔV=100, ΔΠ=1500 are presented in Figure 4. $U_{\infty }$ is about 150 for this set of parameters. The parameters $C_{D}=538.6$ and $x_{0}=0.622$ are obtained by a linear approximation of the value $1/(K\left(\theta =0,r\right)-2\left(1+C_{a}^{0}\right))$ with respect to *r*.

As it can be seen, a simple analytical model achieves a good qualitative description of the nature of the concentration jet. The obvious differences are that there is a zone of reduced concentration on the sides of the jet. This effect occurs due to the fact that the diffusion region arising near the front edge of the particle, beyond the depleted region, spreads downstream, breaks off and is carried away by the flow along with the concentration jet.

**Figure 5:**
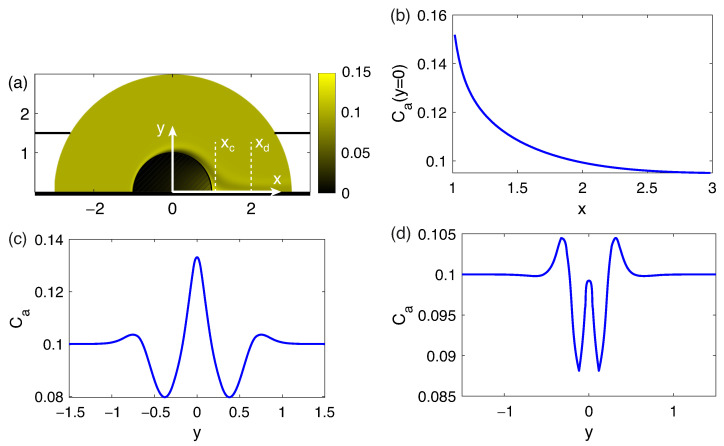
Densities of the third species concentration $C_{a}$ for ΔV=100, ΔΠ=1500 (**a**), concentration density profiles for a numerical modeling along the axis of symmetry (**b**) and in the sections $x_{c}=1.1$ (**c**) and $x_{d}=2$ (**d**), which are indicated in figure (**a**).

The behavior of a positively charged analyte is qualitatively different from the behavior of salt cations and cannot be described by a simple model of the type (25), see Figure 5.

**Figure 6:**
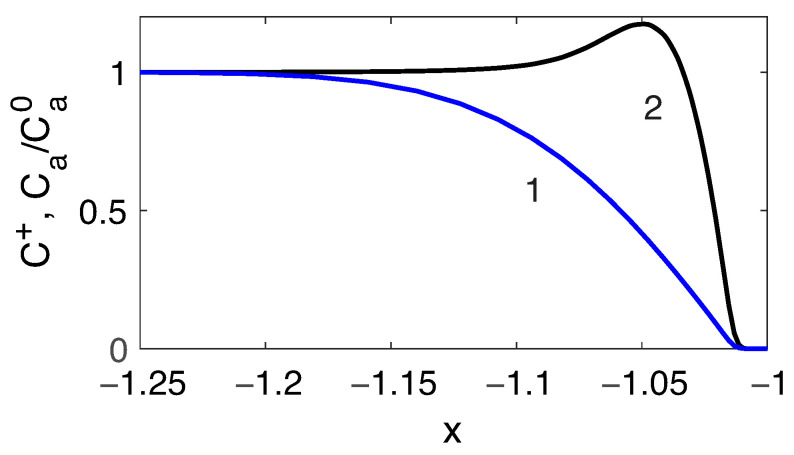
Distributions of the concentration densities of salt cations $C^{+}$ (curve 1) and analyte ions $C_{a}/C_{a}^{0}$ (curve 2) at ΔV=100, ΔΠ = 1500 on the axis of symmetry y=0.

The diffusion layer for the analyte on the front side of the particle also has a different structure in contrast with the similar layer for cations Figure 6, since it has a local maximum concentration of the analyte at the outer boundary of the diffusion layer. This behavior qualitatively corresponds to the results of the ternary electrolyte flow modeling near flat ion-selective surfaces [41].

**Figure 7:**
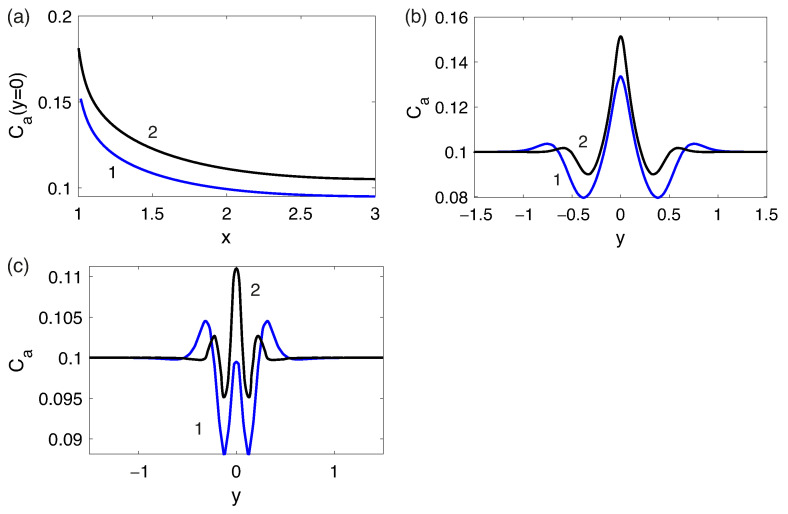
The density of the analyte concentration $C_{a}$ at ΔV=100. The concentration density profiles along the axis of symmetry (**a**) and in sections x=1.1 (**b**) and x=2 (**c**). Curve 1 corresponds to ΔΠ=1500; curve 2, to ΔΠ=4500.

The concentration of the analyte in the jet increases with increasing the pressure, (Figure 7), and the jet itself becomes thinner and diffuses slower downstream.

**Figure 8:**
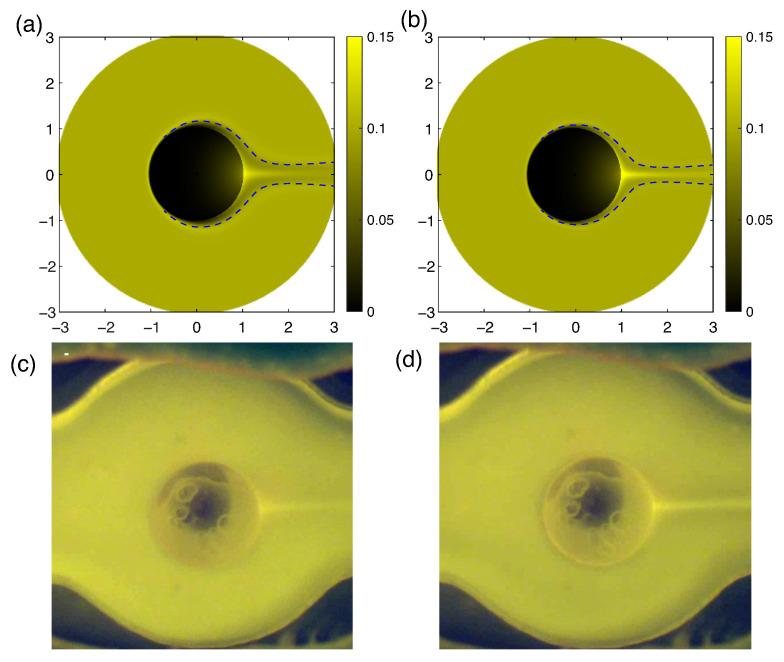
The analyte concentration densities $C_{a}$ at Δ=100 and different values of ΔΠ: (**a**) for ΔΠ=1500, (**b**) for ΔΠ=4500. Dashed blur lines are the visible boundaries of the diffusion layers. The image of Rhodamine at a potential difference 100 V between the electrodes and different flowrates: (**c**) for $\tilde{Q}=0.04$ mL/min, (**d**) for $\tilde{Q}=0.12$ mL/min.

The maximum of the analyte concentration occurs exactly at r=1,θ=0, inside the Debye layer. In order to compare the theoretical results with the experiments, we measured the concentration at the point r=1.1,θ=0. This comparison is presented in Figure 8. The experimental concentration jet appears blurry due to the fact that two-dimensional sections of the axisymmetric formulation are obtained directly with numerical modeling, while the experimental images represent two-dimensional projections of the actual three-dimensional distribution of Rhodamine particles through the entire volume of the cell. For the same reason, the experimental images lack the depleted regions in front of the particle and to the sides of the concentration jet.

The parameter ΔΠ has been selected so that the values of the flowrate coincide in the experiment and the numerical simulation. In contrast, it is not possible to compare the magnitude of the electrical potential drop directly, since in the experimental set-up the electrode chambers are separated from the main chamber with membranes and it is very difficult to determine the potential drop on these membranes. In order to qualitatively evaluate and compare the strengths of external electric fields, the critical ΔV and the difference in electrical potentials at the electrodes at which electroconvection occurs have been considered. The critical potential difference is about 170 V in the experiments, while the critical value of the dimensionless parameter ΔV was about 170 in the numerical simulations.

**Figure 9:**
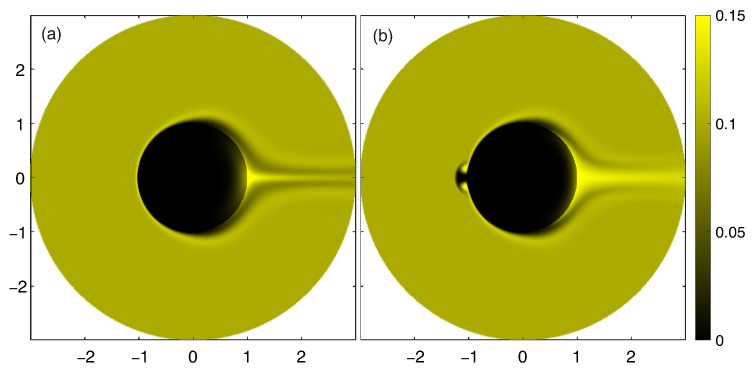
The density of the analyte concentration $C_{a}$ for ΔV=150 and ΔΠ=1500, (**a**) for the primary solution, (**b**) for the secondary solution.

It is possible to observe the occurrence of a secondary solution in the numerical modeling at values of ΔV close to the critical one. The secondary solution is characterized by the presence of a toroidal micro-vortex in the front of the particle, Figure 9. This solution was previously observed in the electrophoresis problem [31]. Whether this solution appears or not depends on initial conditions and perturbations. This regime has not been observed in experiments. Apparently, this is due to its instability: strict axial symmetry is presumably necessary for its maintenance, which is impossible to achieve in experimental installations. With an increase in flowrate, this solution disappears.

### 3.2. Unsteady Regimes

**Figure 10:**
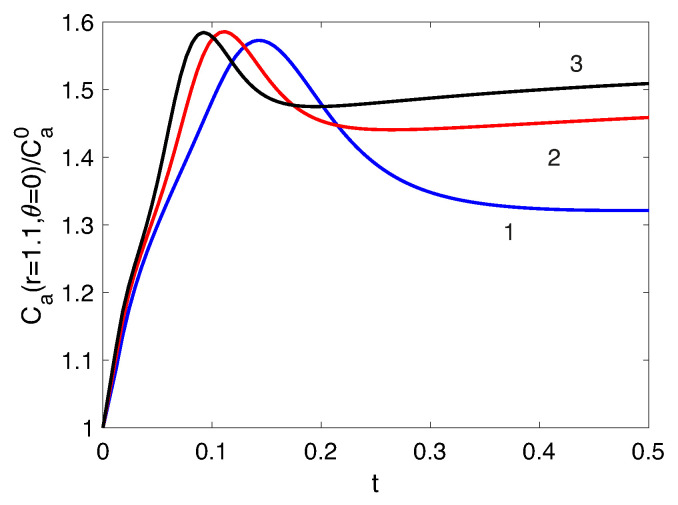
The dependencies of the analyte concentration at the point r=1.1,θ=0 for ΔV=100 and different ΔΠ, 1: ΔΠ=1500, 2: ΔΠ=3000, 3: ΔΠ= 4500.

The presentation of non-stationary regimes is initiated from the description of the processes that occur immediately after the electric field is turned on. At this moment, all the main regions begin to form around the particle. A space charge region is formed; a diffusion zone, which is characterized by a local maximum in the analyte profile (Figure 6), comes next. Dynamically, it appears as a wave of enriched analyte concentration that passes downstream along the entire particle (Video S1 corresponds to ΔV=150, ΔΠ=3000 and Video S2 corresponds to the voltage 140 V and the flowrate 0.08 mL/min in the Supplementary Materials). The dependence of the analyte concentration at the point r=1.1, θ=0 for ΔV=100 and various flow values shows in Figure 10. It can be seen that the concentration temporarily rises above its stationary value: the greater the flowrate is, the faster the stationary regime is established. The figure also shows an increase in the stationary value of the analyte concentration with an increase in flowrate.

The instabilities occur with the increase of ΔV. The primary solution loses stability first at about ΔV=170 for ΔΠ=1500: initial small perturbations near the ion-selective particle grow in time. Following [31], the electric current density through the particle, $j\left(\theta \right)=j^{+}-j^{-}+j_{a}$, is considered in order to observe the evolution of perturbations, where

$$r=1:\;j^{\pm }=\mp C^{\pm }\frac{\partial \Phi }{\partial r}-\frac{\partial C^{\pm }}{\partial r},\;j_{a}=-C_{a}\frac{\partial \Phi }{\partial r}-\frac{\partial C_{a}}{\partial r}.$$

According to [31], j(π/2) and j(π) are chosen for taking a 2D projection of the phase space to characterize the bifurcations. Two steady-state solutions are observed. They are presented in Figure 9 for ΔV=150 and ΔΠ=1500. All solutions are eventually attracted to one of the stationary points (the primary or the secondary one) depending on the initial conditions.

**Figure 11:**
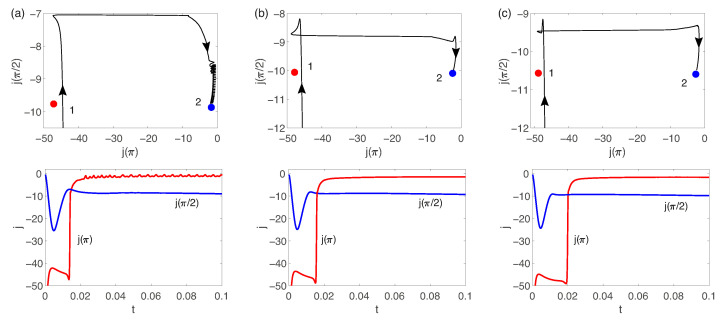
The two attractors in the phase space are two fixed points, corresponding to the primary (point 1) and secondary (point 2) stationary solutions, solid lines correspond to the time evolution, ΔV=170: (**a**) ΔΠ=1500, (**b**) ΔΠ=3000, (**c**) ΔΠ=4500.

The primary point stops attracting solutions with an increase of ΔV, when instability of the primary solution occurs. The solution goes near the primary point, but is eventually attracted for the ΔV=170 to the secondary point with small oscillations (Figure 11a). Increasing the pressure, ΔΠ, stabilizes the flow: the oscillations disappear and the solution spends more time in the vicinity of the primary point (Figure 11b,c).

**Figure 12:**
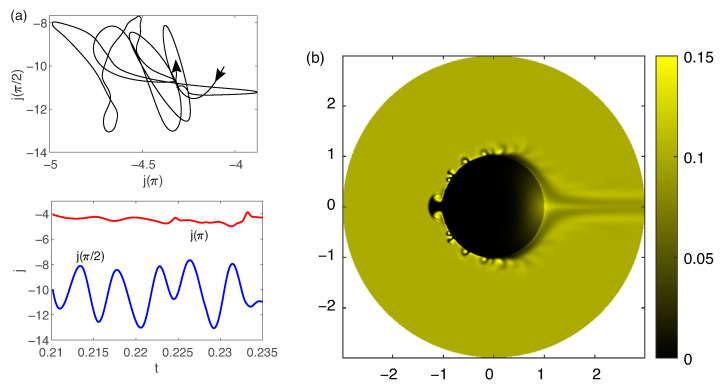
The time evolution (**a**) and a snapshot of the the analyte concentration density $C_{a}$ (**b**) for ΔV=200 and ΔΠ=1500.

Electroconvection occurs when the critical ΔV is exceeded and the secondary solution loses stability as well. The unsteady time evolution is shown in Figure 12. High-amplitude oscillations occur and the regime becomes irregular. The ion distribution corresponds to the data obtained for electrophoresis simulations [31]. The distribution of analyte during electroconvection is qualitatively different, because the analyte accumulates in the center of the electroconvective vortex and the areas of increased and decreased analyte concentrations alternate (Video S3, it corresponds to the parameters in Figure 12). Visually, this picture is similar to electroconvection near a flat ion-selective membrane visualized with Rhodamine [42].

Electroconvection has also been detected experimentally (Video S4, corresponds to the voltage 220 V and to the flowrate 0.12 mL/min), however, the electroconvective vortices were almost invisible. The reason for this, again, could be in deviations from axisymmetry, because the perturbations quickly became three-dimensional while their two-dimensional projections become chaotic.

A simulation of how an axisymmetric electroconvection might appear is possible by considering the three-dimensionality of the concentration distribution in a cylindrical chamber. To do this, the density of the analyte concentration along the *z*-axis can be integrated in the Cartesian coordinate system. Subsequently, from the assumption of a linear dependence of fluorescence on the concentration of dye, we can assume what the propagation of such toroidal electroconvective vortices along the particle would appear as (Video S5 corresponds to parameters in Figure 12). In the real experiment, the fluorescence does not linearly depend on the concentration of Rhodamine [19], and thus more in-depth investigations are necessary for a detailed comparison with the numerical simulation.

## 4. Conclusions

The results of the numerical simulation and the experimental investigation of the analyte behavior near the ion-selective microparticle in the presence of electrokinetically and pressure-driven flow are presented in this paper. The fluorescent agent Rhodamine-6G was used as an analyte, which was diluted in a potassium chloride solution. A special cell was developed for the investigation. The cell for the numerical simulation had a spherical form with a round inlet and outlet, and an ion-selective microparticle anchored in its center. The simulations revealed the appearance of a concentration jet for both the salt ions and for the analyte. The salt jet behavior might be described by a simple model of an axisymmetric wake behind a body of revolution in an axial flow. The analyte jet exhibits a more complex structure and warrants a more thorough investigation. The degree of the concentration increased with an increase in the pressure-driven flow, while instabilities appeared with an increase in the electric field strength.

The experimental cell was constructed through 3D photopolymer printing and contained a cylindrical chamber in the middle for proper visualization. The experimental results confirmed the presence of the analyte concentration jet and the occurrence of the electroconvection for a sufficiently large external electric field.

The results above provide a clearer view on the superconcentration phenomenon which allows researchers to design, improve and optimize microdevices based on membrane technology. Although detection and preconcentration problems are uncommon for membranes, they are actively being studied. The corresponding devices are known as membrane sensors, which could be efficiently used for simplifying and improving the accuracy of chemical and medical analyzes. Thus, the results of this paper provide important information required for further studies on the efficiency of such devices.

It is important to note that the designed cell might also work as a micropump and a micromixer, which has been demonstrated in our previous studies. The present investigation allows us to conclude that the analyte superconcentration might be observed in the cell once the parameters are specifically chosen. The search for such parameter values remains the topic of future investigations.

## Data Availability

The datasets generated during and/or analysed during the current study are available from the corresponding author upon reasonable request.

## Supplementary Materials

The following supporting information can be downloaded at: https://www.mdpi.com/article/10.3390/membranes13050503/s1, Video S1: The density of the analyte concentration evolution for ΔV=150, ΔΠ=3000; Video S2: The experimental video for the 140 V voltage and 0.08 mL/min flowrate; Video S3: The density of the analyte concentration evolution for ΔV=200, ΔΠ=1500; Video S4: The experimental video for the 220 V voltage and 0.12 mL/min flowrate; Video S5: The mean along the z-axis density of the analyte concentration evolution for ΔV=200, ΔΠ=1500.

## Abbreviations

The following abbreviations are used in this manuscript:

${\tilde{r}}_{0}$	the radius of the ion-selective granule, which is taken as a characteristic length;
${\tilde{r}}_{0}^{2}/\tilde{D}$	the characteristic time;
$\tilde{D}/{\tilde{r}}_{0}$	the characteristic velocity;
$\tilde{\mu }$	the liquid viscosity, which is taken as a characteristic dynamic value;
$\tilde{\mu }\tilde{D}/{\tilde{r}}_{0}^{2}$	the characteristic stress;
${\tilde{\Phi }}_{0}=\tilde{R}\tilde{T}/\tilde{F}$	the thermal potential, which is taken as a characteristic voltage;
${\tilde{C}}_{\infty }$	the concentration of cations in the reservoir on the outside of the inlet;
$\tilde{D}\tilde{F}{\tilde{C}}_{\infty }/{\tilde{r}}_{0}$	the characteristic electric current.

## References

[1] Kumar S., Maniya N., Wang C., Senapati S., Chang H.-C. (2023). Quantifying PON1 on HDL with Nanoparticle-Gated Electrokinetic Membrane Sensor for Accurate Cardiovascular Risk Assessment. Nat. Commun..

[2] Ren X., Ellis B.W., Ronan G., Blood S.R., DeShetler C., Senapati S., March K.L., Handberg E., Anderson D., Pepine C. (2021). A Multiplexed Ion-Exchange Membrane-Based MiRNA (MIX·miR) Detection Platform for Rapid Diagnosis of Myocardial Infarction. Lab. Chip..

[3] Ramshani Z., Zhang C., Richards K., Chen L., Xu G., Stiles B.L., Hill R., Senapati S., Go D.B., Chang H.-C. (2019). Extracellular Vesicle MicroRNA Quantification from Plasma Using an Integrated Microfluidic Device. Commun. Biol..

[4] Chen Q., Liu X., Lei Y., Zhu H. (2022). An Electrokinetic Preconcentration Trapping Pattern in Electromembrane Microfluidics. Phys. Fluids.

[5] Ouyang W., Ye X., Li Z., Han J. (2018). Deciphering Ion Concentration Polarization-Based Electrokinetic Molecular Concentration at the Micro-Nanofluidic Interface: Theoretical Limits and Scaling Laws. Nanoscale.

[6] Chang H.-C., Yeo L.Y. (2010). Electrokinetically Driven Microfluidics and Nanofluidics.

[7] Chang H.-C., Yossifon G., Demekhin E.A. (2012). Nanoscale Electrokinetics and Microvortices: How Microhydrodynamics Affects Nanofluidic Ion Flux. Annu. Rev. Fluid Mech..

[8] Ben Y., Demekhin E.A., Chang H.-C. (2004). Nonlinear Electrokinetics and “superfast” Electrophoresis. J. Colloid Interface Sci..

[9] Rubinstein I., Zaltzman B. (2000). Electro-Osmotically Induced Convection at a Permselective Membrane. Phys. Rev. E.

[10] Pismenskaya N.D., Nikonenko V.V., Belova E.I., Lopatkova G.Y., Sistat P., Pourcelly G., Larshe K. (2007). Coupled Convection of Solution near the Surface of Ion-Exchange Membranes in Intensive Current Regimes. Russ. J. Electrochem..

[11] Demekhin E.A., Shelistov V.S., Polyanskikh S.V. (2011). Linear and Nonlinear Evolution and Diffusion Layer Selection in Electrokinetic Instability. Phys. Rev. E.

[12] Demekhin E.A., Nikitin N.V., Shelistov V.S. (2014). Three-Dimensional Coherent Structures of Electrokinetic Instability. Phys. Rev. E.

[13] Mareev S.A., Nebavskiy A.V., Nichka V.S., Urtenov M.K., Nikonenko V.V. (2019). The Nature of Two Transition Times on Chronopotentiograms of Heterogeneous Ion Exchange Membranes: 2D Modelling. J. Membr. Sci..

[14] Butylskii D.Y., Mareev S.A., Pismenskaya N.D., Apel P.Y., Polezhaeva O.A., Nikonenko V.V. (2018). Phenomenon of Two Transition Times in Chronopotentiometry of Electrically Inhomogeneous Ion Exchange Membranes. Electrochim. Acta.

[15] Nikonenko V.V., Mareev S.A., Pismenskaya N.D., Uzdenova A.M., Kovalenko A.V., Urtenov M.K., Pourcelly G. (2017). Effect of Electroconvection and Its Use in Intensifying the Mass Transfer in Electrodialysis (Review). Russ. J. Electrochem..

[16] Liang Y.Y., Fimbres Weihs G.A., Fletcher D.F. (2018). CFD study of the effect of unsteady slip velocity waveform on shear stress in membrane systems. Chem. Eng. Sci..

[17] Kim S.J., Wang Y.-C., Lee J.H., Jang H., Han J. (2007). Concentration Polarization and Nonlinear Electrokinetic Flow near a Nanofluidic Channel. Phys. Rev. Lett..

[18] de Jong J., Lammertink R.G.H., Wessling M. (2006). Membranes and microfluidics: A review. Lab. Chip..

[19] Wang Y.-C., Stevens A.L., Han J. (2005). Million-Fold Preconcentration of Proteins and Peptides by Nanofluidic Filter. Anal. Chem..

[20] Wang S.-C., Lai Y.-W., Ben Y., Chang H.-C. (2004). Microfluidic Mixing by Dc and Ac Nonlinear Electrokinetic Vortex Flows. Ind. Eng. Chem. Res..

[21] Wang S.-C., Wei H.-H., Chen H.-P., Tsai M.-H., Yu C.-C., Chang H.-C. (2008). Dynamic Superconcentration at Critical-Point Double-Layer Gates of Conducting Nanoporous Granules Due to Asymmetric Tangential Fluxes. Biomicrofluidics.

[22] Mishchuk N.A., Heldal T., Volden T., Auerswald J., Knapp H. (2011). Microfluidic Pump Based on the Phenomenon of Electroosmosis of the Second Kind. Microfluid. Nanofluid.

[23] Schiffbauer J., Ganchenko G., Nikitin N., Alekseev M., Demekhin E. (2021). Novel Electroosmotic Micromixer Configuration Based on Ion-selective Microsphere. Electrophoresis.

[24] Chen H.-P., Tsai C.-C., Lee H.-M., Wang S.-C., Chang H.-C. (2013). Selective dynamic concentration of peptides at poles of cation-selective nanoporous granules. Biomicrofluidics.

[25] Polezhaev P., Bellon T., Vobecka L., Slouka Z. (2021). Molecular sieving of alkyl sulfate anions on strong basic gel-type anion-exchange resins. Sep. Purif. Technol..

[26] Mareev S., Gorobchenko A., Ivanov D., Anokhin D., Nikonenko V. (2022). Ion and Water Transport in Ion-Exchange Membranes for Power Generation Systems: Guidelines for Modeling. Int. J. Mol. Sci..

[27] Schiffbauer J., Leibowitz N., Yossifon G. (2015). Extended Space Charge near Nonideally Selective Membranes and Nanochannels. Phys. Rev. E.

[28] Schiffbauer J., Demekhin E., Ganchenko G. (2020). Transitions and Instabilities in Imperfect Ion-Selective Membranes. Int. J. Mol. Sci..

[29] Rubinstein I., Shtilman L. (1979). Voltage against Current Curves of Cation Exchange Membranes. J. Chem. Soc. Faraday Trans. 2.

[30] Ganchenko G.S., Frants E.A., Shelistov V.S., Nikitin N.V., Amiroudine S., Demekhin E.A. (2019). Extreme Nonequilibrium Electrophoresis of an Ion-Selective Microgranule. Phys. Rev. Fluids.

[31] Ganchenko G.S., Frants E.A., Amiroudine S., Demekhin E.A. (2020). Instabilities, Bifurcations, and Transition to Chaos in Electrophoresis of Charge-Selective Microparticle. Phys. Fluids.

[32] Schnitzer O., Yariv E. (2012). Dielectric-Solid Polarization at Strong Fields: Breakdown of Smoluchowski’s Electrophoresis Formula. Phys. Fluids.

[33] Demekhin E.A., Nikitin N.V., Shelistov V.S. (2013). Direct Numerical Simulation of Electrokinetic Instability and Transition to Chaotic Motion. Phys. Fluids.

[34] Nikitin N. (2006). Third-order-accurate Semi-implicit Runge–Kutta Scheme for Incompressible Navier–Stokes Equations. Int. J. Numer. Meth. Fluids.

[35] Frants E.A., Ganchenko G.S., Shelistov V.S., Amiroudine S., Demekhin E.A. (2018). Nonequilibrium Electrophoresis of an Ion-Selective Microgranule for Weak and Moderate External Electric Fields. Phys. Fluids.

[36] Schnitzer O., Zeyde R., Yavneh I., Yariv E. (2013). Weakly Nonlinear Electrophoresis of a Highly Charged Colloidal Particle. Phys. Fluids.

[37] Dukhin S.S. (1991). Electrokinetic Phenomena of the Second Kind and Their Applications. Adv. Colloid Interface Sci..

[38] Zaltzman B., Rubinstein I. (2007). Electro-Osmotic Slip and Electroconvective Instability. J. Fluid. Mech..

[39] Schlichting H., Gersten K. (2017). Boundary-Layer Theory.

[40] Amiroudine S., Demekhin E.A., Ganchenko G.S., Shelistov V.S., Frants E.A. (2022). Instability of a Salt Jet Emitted from a Point Source in an External Electric Field. Phys. Fluids.

[41] Kovalenko A.V., Khromykh A.A., Urtenov M.K. (2014). Decomposition of the Two-Dimensional Nernst–Planck–Poisson Equations for a Ternary Electrolyte. Dokl. Math..

[42] Kovalenko A.V., Wessling M., Nikonenko V.V., Mareev S.A., Moroz I.A., Evdochenko E., Urtenov M.K. (2021). Space-Charge Breakdown Phenomenon and Spatio-Temporal Ion Concentration and Fluid Flow Patterns in Overlimiting Current Electrodialysis. J. Membr. Sci..

