# Supplementary figures and images for: Electrokinetic and Electroconvective Effects in Ternary Electrolyte Near Ion-Selective Microsphere
Source: Membranes (Basel). 2023 May 10;13(5):503. doi: 10.3390/membranes13050503 (PMC10221117; doi:10.3390/membranes13050503)

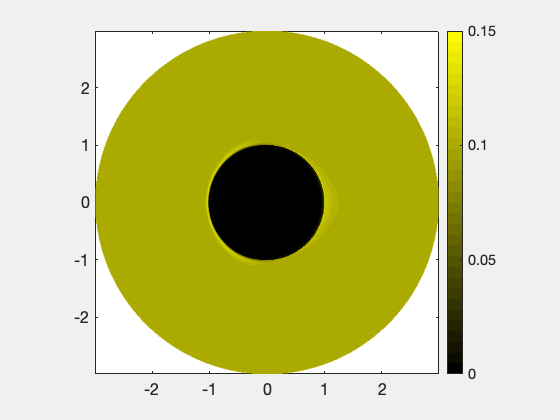

Supplement: Supplementary file 1 [file membranes-13-00503-s001.zip › animations/Video_S1.gif]

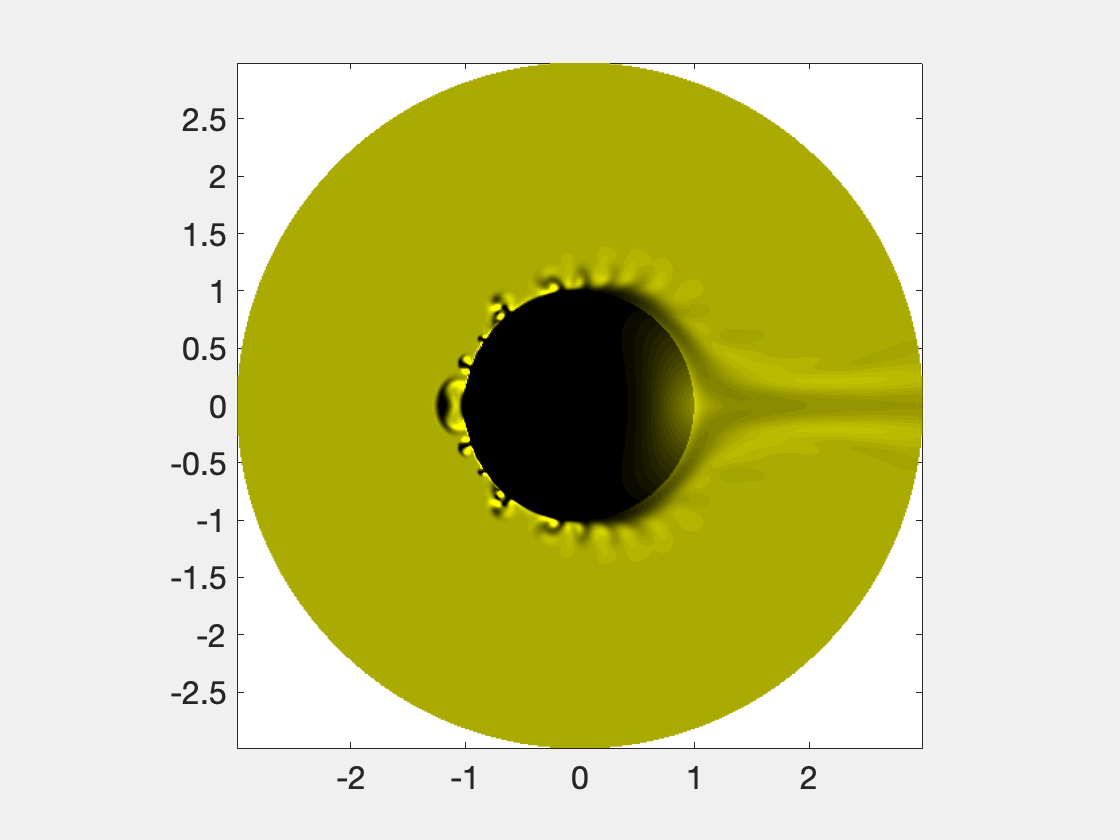

Supplement: Supplementary file 1 [file membranes-13-00503-s001.zip › animations/Video_S3.gif]

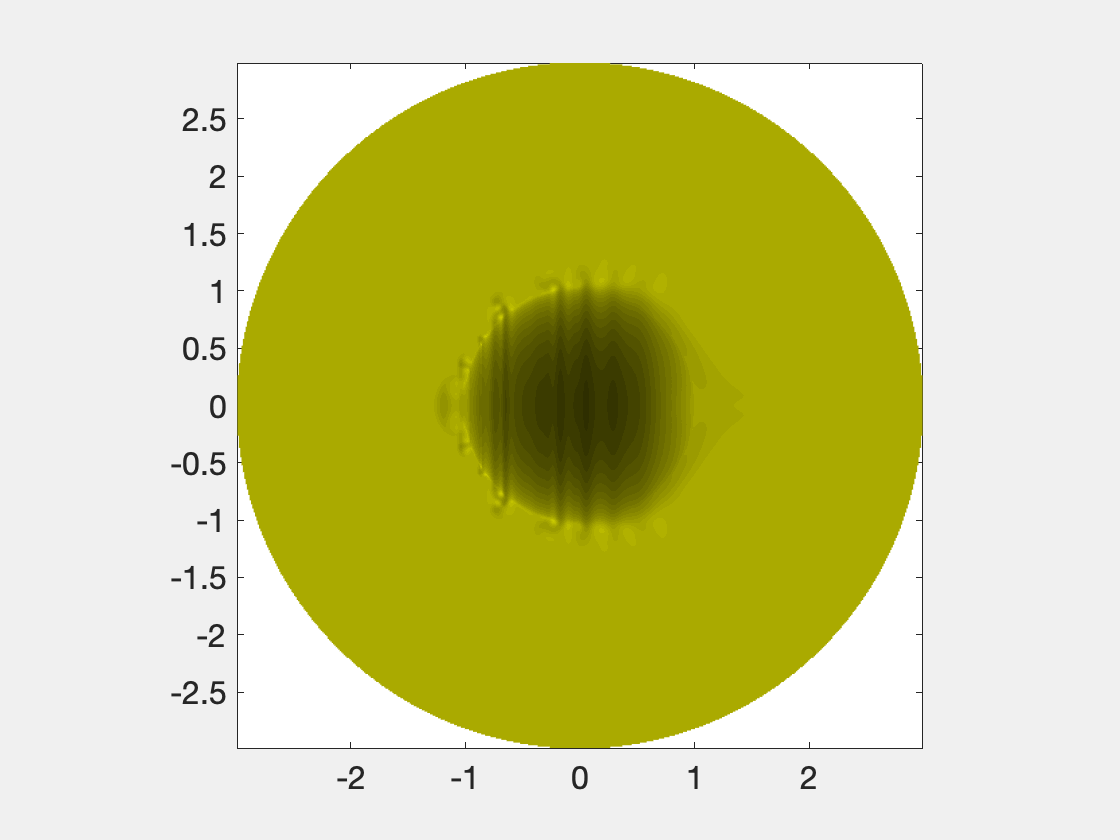

Supplement: Supplementary file 1 [file membranes-13-00503-s001.zip › animations/Video_S5.gif]
